# Exploring the Relationship Between Human Social Deprivation and Animal Surrender to Shelters in British Columbia, Canada

**DOI:** 10.3389/fvets.2021.656597

**Published:** 2021-03-10

**Authors:** Lexis H. Ly, Emilia Gordon, Alexandra Protopopova

**Affiliations:** ^1^Animal Welfare Program, Faculty of Land and Food Systems, University of British Columbia, Vancouver, BC, Canada; ^2^The British Columbia Society for the Prevention of Cruelty to Animals, Vancouver, BC, Canada

**Keywords:** animal shelter, relinquishment, animal welfare, one welfare, social determinants, social vulnerability

## Abstract

Previous studies identify owner-related issues, such as cost and housing, as common reasons for relinquishment of companion animals to animal shelters. It is likely that the burden of surrendering for owner-related reasons falls on those who are socially vulnerable (e.g., low income, unemployed); however, very few studies have assessed social determinants as a predictor of animal relinquishment. The present study used the Canadian Index of Multiple Deprivation (CIMD), which uses four factors of social vulnerability (Ethnocultural Composition, Economic Dependency, Residential Instability, and Situational Vulnerability) to predict risk of surrender for various reasons, of various species and breeds, and of various health statuses across British Columbia, Canada (*n* = 29,236). We found that CIMD factors predicted increased risk of surrender across many shelter variables. For further understanding of differences between areas in the province, the present study also analyzed the relationship between CIMD factors and animal surrender variables in two areas of interest: Metro Vancouver (*n* = 3,445) and Kamloops (*n* = 2,665), and plotted these relationships on a geospatial scale. We found that there were some similarities across areas, such as Situational Vulnerability predicting increased odds of surrendering pit bull-labeled dogs vs. all other dog breeds. There were also differences in predictors of animal surrender variables, suggesting that provision of animal services, such as veterinary care, for vulnerable groups may be specific to location. For example, whereas Ethnocultural Composition predicted increased risk of owner surrender for multiple owner-related reasons in Metro Vancouver, these same reasons for surrender were predicted by Residential Instability in Kamloops, indicating demographic differences that affect animal shelter service use. The results of this research validate the use of geospatial analysis to understand relationships between human vulnerability and animal welfare, but also highlight the need for further interventions in marginalized populations to increase retention of animals.

## Introduction

The close relationship of humans and companion animals means that the two populations often share similar physical and social conditions (1); however, human and animal services, such as shelters for humans and animals experiencing homelessness, often remain segregated (2). Recently, the One Health framework—which reflects the interconnected nature of the health of humans, animals, and the environment—has become more popular, although it typically focuses on threats to the health of the three aforementioned facets, such as zoonotic diseases or antimicrobial resistance (3). In response, the One Welfare framework was created as a unifying concept to include both physical and psychosocial impacts of human animal relationships, and how improvement of one can ultimately benefit the other (4). Pet ownership falls under the One Welfare framework, as previous studies report various physical and mental health benefits to owning a companion animal (5–7).

Pet ownership is not equal across human groups. Previous studies have found pet ownership to be associated with social factors such as housing type, house ownership, income, education, family composition, and urban vs. rural residency, among others (8–10). Studies report owner-related issues (including housing issues, owner health issues, cost) as a major reason for surrender [for a review on dog surrender, see (11)]. Miller et al. (12) reported 87.2% of cat surrenders in an Australian shelter being due to owner-related reasons. The significance of owner-related reasons for surrender indicates that animal relinquishment may be a One Welfare issue as well, as owner factors (such as poor health, housing issues, and low socioeconomic status) may create greater risk of relinquishing their animal. Previous research found that areas of high social vulnerability predicted both dog “hot spots” —areas from which high amounts of dogs were relinquished—and indicated higher intake of dogs with health issues and social neglect (1). Another study found that areas of high intake of stray dogs overlapped with areas of child maltreatment, which may indicate that such communities face multiple human and animal related challenges that may be driven by social deprivation (13).

Whereas, the aforementioned studies all identified relationships between human social determinants and animal shelter-related variables, none of these studies have more broadly analyzed community-level social determinants with various aspects of relinquishment, such as the species surrendered, the reason for surrender, or the health status of the animal. Given the prevalence of owner-related reasons for surrender, the relationship between human deprivation and risk of surrender across the given reasons for surrender are an area of interest. Additionally, pet ownership and surrender has been studied extensively in dogs and cats, although the growing popularity of small animals, rabbits, and exotic pets in North America warrants investigation (14, 15). Geospatial analysis lends itself well to comparisons of various geographic areas; however, previous studies in animal welfare often study one location of interest (1, 13). The aim of the present study was to explore the relationship between community-level social deprivation and owner surrender characteristics to animal shelters in British Columbia, Canada. The analysis took place on a province-wide scale across British Columbia, as well as a focused analysis of two demographically different areas of the province: Metro Vancouver and Kamloops. We utilized data from the British Columbia Society for the Prevention of Cruelty of Animals (BC SPCA), which has 36 animal shelter locations across the province. We used statistical analysis of data from the Canadian Index of Multiple Deprivation (CIMD), as well as geospatial analysis to demonstrate the relationship between social determinants and animal relinquishment.

## Materials and Methods

### Data

This study protocol was reviewed and approved by the University of British Columbia's Behavioral Research Ethics Board (H20-02704). Permission for data collection was received by the BC SPCA. This retrospective study utilized the publicly available CIMD, which uses various measures of social well-being categorized into four factors to create nation-wide and region-specific scales (16). The CIMD uses Canadian census data to determine deprivation based on dissemination area. A dissemination area is a unit of area used by the Canadian census that contains around 400–700 people; it is made up of one or more dissemination blocks [a unit of area that is bound by standard geographic areas such as roads or boundaries; (16)]. The data exists in both raw numeric scores and quintile scores. A higher score indicates greater deprivation based on the indicators for each particular dimension (16). The four dimensions of the CIMD are Ethnocultural Composition (EC), Situational Vulnerability (SV), Economic Dependency (ED), and Residential Instability (RI). Table 1 shows corresponding indicators for each of the four dimensions for British Columbia.

**Table 1:** The four dimensions of multiple deprivation and corresponding indicators for British Columbia (2016).

**Ethnocultural composition**	**Situational vulnerability**	**Economic dependency**	**Residential instability**
Proportion of population who self-identify as a visible minority	Proportion of population that identifies as aboriginal	Proportion of population participating in the labor force (>15yrs)^*^	Proportion of dwellings that are apartment buildings
Proportion of population that is foreign-born	Proportion of population aged 25–64 without a high school diploma	Proportion of population aged 65+	Proportion of persons living alone
Proportion of population with no knowledge of either official language (linguistic isolation)	Proportion of dwellings needing major repairs	Ratio of employment to population^*^	Proportion of dwellings that are owned^*^
Proportion of population who are recent immigrants (arrived in 5 years prior to Census)	Proportion of population that is low-income	Dependency ratio (population 0–14 and 65+ divided by population 15–64)	Proportion of population who moved within the past 5 years
	Proportion of single parent families		

* *Indicates reverse-coded measures. Data is taken from the 2016 Census of Population by Statistics Canada*.

In order to link the person's postal code with the Statistics Canada's standard geographic areas for which the CIMD factors are measured, the Postal Code Conversion File (PCCF) was used (17). In some rural, low-population areas of British Columbia, there are some postal codes which span a large area and a few cases where the postal code spans multiple dissemination areas. To simplify the data, the first dissemination area corresponding with these postal codes was used. To verify the use of the chosen dissemination area score, we calculated the Pearson correlation coefficient (for raw numeric scores) and weighted Cohen's Kappa (for quintile scores) between the chosen score and the mean scores across each CIMD factor (Ethnocultural Composition R = 0.99, Kappa = 0.98; Economic Dependency R = 0.99, Kappa = 0.97; Situational Vulnerability R = 0.98, Kappa = 0.97; Residential Instability R = 0.99, Kappa = 0.97).

Incoming animal data were requested from all 36 animal shelter branches of the British Columbia Society for the Prevention of Cruelty of Animals from September 1, 2016 to August 31, 2020. The first animal shelter variable of interest was the reason for surrender, which were grouped into 10 distinct categories by the shelter data collection software: Personal Issues (including divorce, pregnancy/recent birth, or jail), No Longer Wanted (the animal was a gift or is unwanted), Housing Issues (including eviction, complaints from neighbors, being unable to find pet-friendly housing), Guardian Health (including owner sickness or injury, allergies to pet, death of owner), Feral/Free-roaming (surrender of a feral or free-roaming animal), Can't Afford (including being unable to pay for veterinary costs, maintenance costs, or impound fees), Behavior (including animal behavioral issues such as aggression, digging/chewing, house training issues), Animal Health (including animal injuries, sickness, or animal pregnancy), Abandoned (by a friend, relative, or tenant), and Too Many (including unwanted litters, and having too many animals in the home), which was used as the reference category. The reference category in each shelter variable is used as a category of comparison for the rest of the categories. The reference category for each shelter variable is the most commonly reported category. Another shelter variable of interest was species, which was grouped into six categories: small animal, rabbit, puppy, kitten, exotic, dog, and cat (which was used as a reference category). Finally, the last multi-category variable of interest was the animals' physical and behavioral health status upon intake, which was recorded based on the Asilomar Accords (18) and includes Healthy (which was used as the reference category), Treatable-Manageable, Treatable-Rehabilitatable, and Unhealthy-Untreatable. Binary variables of interest included whether the animal was spayed/neutered upon entering the shelter (compared to sexually intact animals), which was analyzed for cats, dogs, kittens, and puppies only, and whether or not the animal was a pit bull-labeled dog (compared to all other labeled dog breeds), which was analyzed for dogs and puppies only. We had a particular interest in pit bull-labeled dogs due to long-debated links between this breed type, race, and poverty (19). The term “pit bull-labeled” rather than “pit bull type” is used because of previous literature which shows that shelter staff frequently misidentify dogs belonging to this group (20).

The raw shelter data files contained 76,991 observations (2016–2017: *n* = 22,791; 2017–2018: *n* = 22,245; 2018–2019: *n* = 21,254; 2019–2020: *n* = 16,646). In order to assess the data geospatially within the province, all entries with postal codes outside of British Columbia were excluded. Observations were also removed if they did not have any location data, as they could not be related to the CIMD scale. Data were subset into the method of intake (Owner Surrender, Stray, and Humane Officer Seized/Surrendered). For owner surrendered animals, shelter-related variables of interest included reason for surrender, species, spay/neuter status upon intake and dog pit bull label.

### Analysis

For shelter variables which had two possible categories (spay/neuter upon intake, pit bull label), a logistic regression was performed to examine the relation between the four dimensions of the CIMD and the shelter variable. Relationships between the categorical shelter variables (reason for surrender, species, health status) and CIMD dimensions were assessed through a multinomial logistic regression. Relationships were considered statistically significant at *p* < 0.05. Due to the exploratory nature of this study, no formal adjustment for multiplicity of testing was included in order to allow for subsequent studies to confirm or refute possible connections (21). All statistical analysis was undertaken in R Studio.

Coordinates of the owner addresses given at intake were plotted onto a map of British Columbia's dissemination areas. Because the area of British Columbia is so large, we chose to focus on two regions of the province to demonstrate spatial relationships (Metro Vancouver, and Kamloops and its surrounding area). Results from the statistical analysis across all of British Columbia were used to discuss which relationships between CIMD factors and shelter-related data. For the Metro Vancouver and Kamloops analysis, several statistically significant results were plotted geospatially for descriptive purposes in order to visually demonstrate differences between locations within the province. Metro Vancouver was selected because it is the most populous location in BC. As a comparison to Vancouver, Kamloops was selected because of its lower population, differing Ethnocultural Composition, and a more rural environment. Additionally, Kamloops is one of three BC municipalities where the BC SPCA is conducting focused community assessments thus allowing for the practical application of collected data.

### Study Locations

In 2016, the population of British Columbia was ~4.6 million people. The majority of the population (64%) are white, followed by East and Southeast Asians (18%, 18). The reported Indigenous population in 2016 was ~300,000 people. The majority of immigrants to British Columbia moved to the province within the past 30 years. Since 1981, the most common birthplace of immigrants to British Columbia was China, followed by India and the Philippines (22).

Metro Vancouver is a metropolitan area, which consists of a large urban area (the City of Vancouver) and surrounding “urban fringes” (23). In 2016, the reported population of Metro Vancouver was ~2.5 million people (24). The City of Vancouver is located on the homelands of the Musqueam, Squamish, and Tsleil-Waututh First Nations (25). In 2016, 2.5% of the Metro Vancouver population reported Indigenous ancestry, and Indigenous population has seen a net growth since 1996 (24). The largest pan-ethnic groups in Metro Vancouver are white (49%) followed by East Asian (23%). Under Statistics Canada's definition of “visible minority” (neither white nor Indigenous), the population of visible minorities in Metro Vancouver grew by 110% between 1996 and 2016 (24). Across Metro Vancouver, 36% of households are reported as rentals, although this proportion increases to 53% of households when looking specifically at the City of Vancouver (25).

Kamloops is a city located in the south-central area of British Columbia that reported an estimated population of 100,000 in 2016 (26). The proportion of visible minorities is significantly smaller compared to Metro Vancouver, with the total visible minority population reported as 8% in 2016, with South Asians making up 36% of the visible minority population. Kamloops has a higher proportion of Indigenous residents (10%) compared to Metro Vancouver (26). Unlike Metro Vancouver, the largest influx of immigrants to Kamloops was prior to 1981. Kamloops also has a lower proportion of renters compared to Metro Vancouver [28%; (21)].

## Results

### British Columbia

**Figure 1:**
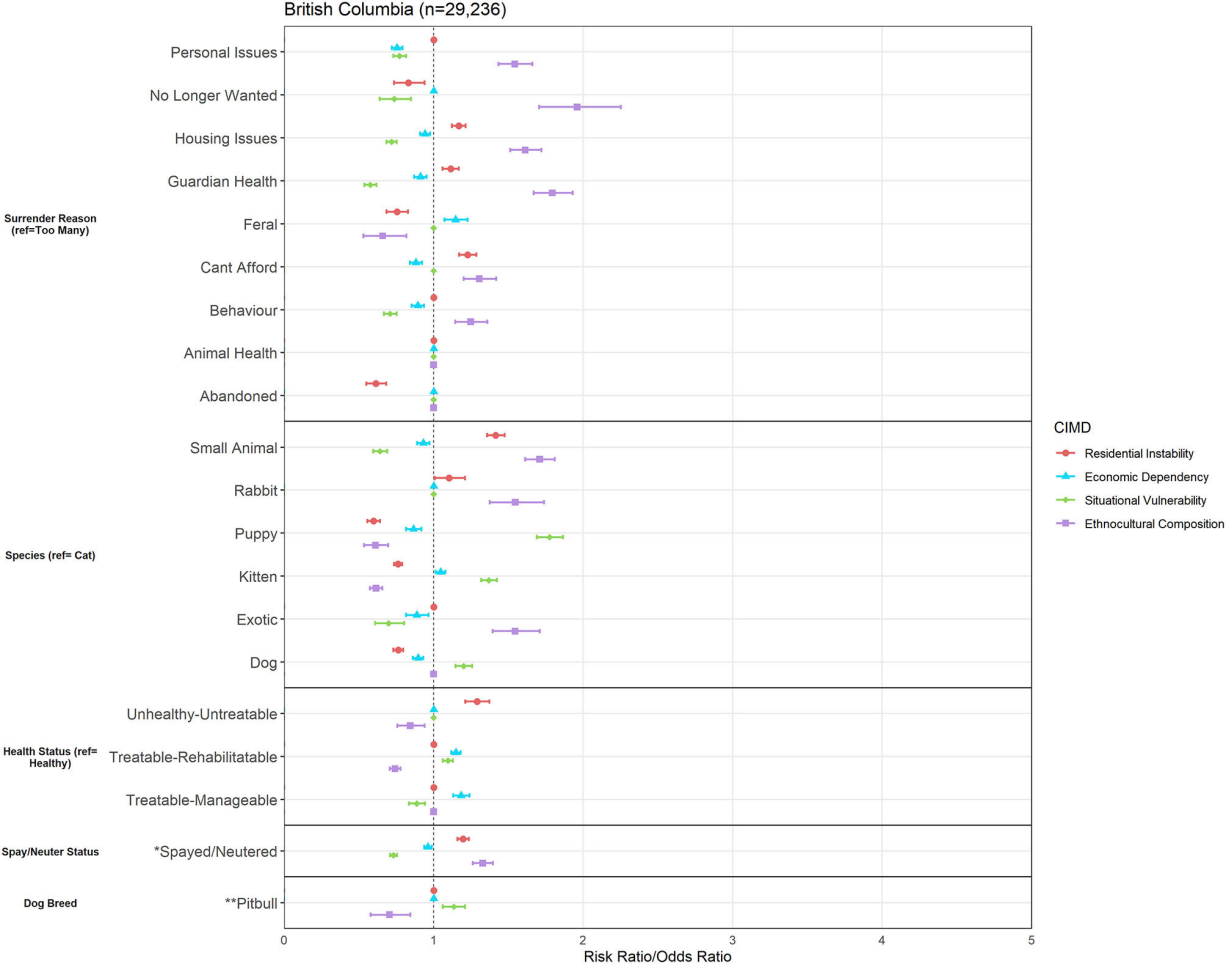
Association between Canadian Index of Multiple Deprivation (CIMD) factors and owner surrender variables across British Columbia (*n* = 29,236). Results of logistic regression models for shelter variables by increasing CIMD factor scores are shown. Data are presented by risk ratios and their 95% confidence interval (bars); *p* < 0.05 when 95% confidence interval does not cross the vertical dotted line. Non-significant results are represented by a risk ratio of 1 with no CI. A risk ratio >1 suggests an increased risk of that shelter variable category as you increase in deprivation, while a risk ratio <1 suggests a reduced risk. *denotes data from dogs, cats, kittens, and puppies only (*n* = 25, 075) and is represented in odds ratios (and 95% CI). **Denotes data from dogs and puppies only (*n* = 6,516) and is represented in odds ratios (and 95% CI).

After the data cleaning, the total number of observations for Owner Surrender data for all of British Columbia (2016–2020) was *n* = 29,236. Results of the multinomial logistic regression models showed that there were various associations between the CIMD factors and owner surrender variables, as seen in Figure 1.

#### Ethnocultural Composition

Ethnocultural Composition predicted risk of surrendering for various owner-related reasons for surrender. A one-unit increase in Ethnocultural Composition increased risk of surrendering for personal issues by 1.54 times (95% CI, 1.43–1.66), housing issues by 1.61 times (95% CI, 1.51–1.72), guardian health by 1.79 times (95% CI, 1.67–1.93) and being unable to afford the animal by 1.31 times (95% CI, 1.20–1.42) in comparison to surrendering for having too many animals. Increase in Ethnocultural Composition predicted increased risk of surrendering for animal-related reasons such as the animals' behavior (RR = 1.25, 95% CI, 1.14–1.36), but predicted decreased risk of surrendering for reasons of the animal being feral/free-roaming (RR = 0.66, 95% CI, 0.53–0.82). Ethnocultural Composition also predicted the greatest increase in risk of surrendering for reasons of no longer wanting the animal (RR = 1.96, 95% CI, 1.71–2.25) across all four CIMD factors.

In comparison to surrendering cats, an increase in Ethnocultural Composition increased risk of surrendering rabbits and small animals by 1.54 (95% CI, 1.38–1.74) and 1.71 (95% CI, 1.61–1.81) times, respectively. Increase in Ethnocultural Composition predicted decreased risk of surrendering kittens and puppies in comparison to cats (kitten RR = 0.61, 95% CI, 0.57–0.66; puppy RR = 0.61, 95% CI, 0.54–0.70). Ethnocultural Composition predicted decreased risk of surrendering animals which were Unhealthy-Untreatable or Treatable-Rehabilitatable compared to those considered Healthy (Unhealthy-Untreatable RR = 0.84, 95% CI, 0.76–0.94; Treatable-Rehabilitatable RR = 0.74, 95% CI, 0.71–0.78). The odds of surrendering an animal that is spayed or neutered (relative to not spayed or unneutered) were 1.33–1 for every unit increase in Ethnocultural Composition (95% CI, 1.26–1.40), and odds of surrendering a pit bull-labeled dog compared to all other dogs was decreased (OR = 0.70, 95% CI, 0.58–0.84).

#### Situational Vulnerability

An increase in Situational Vulnerability did not predict increased risk of any reasons for surrender compared to surrendering for having too many animals. Increase in Situational Vulnerability lowered risk of surrendering for reasons of animal behavior (RR = 0.71, 95% CI, 0.67–0.75), guardian health issues (RR = 0.58, 95% CI, 0.54–0.62), housing issues (RR = 0.72, 95% CI, 0.69–0.75), no longer wanting the animal (RR = 0.62, 95% CI, 0.64–0.85), and for personal issues (RR = 0.77, 95% CI, 0.73–0.82) in comparison to surrendering for having too many animals. A one-unit increase of Situational Vulnerability increased risk of surrendering kittens and puppies by 1.37 (95% CI, 1.32–1.42) and 1.78 (95% CI, 1.69–1.86) times, respectively, in comparison to cat surrenders. Among dogs and puppies, for each one-unit increase in Situational Vulnerability, odds of surrendering a pit bull-labeled dog were 1.13 times larger than surrendering any other dog breed (95% CI, 1.06–1.21).

#### Economic Dependency

A one-unit increase in Economic Dependency increased risk of surrender due to the animal being feral/free-roaming by 1.15 times (95% CI, 1.07–1.23). Increase in Economic Dependency reduced risk of surrendering for reasons of animal behavior (RR = 0.89, 95% CI, 0.85–0.94), being unable to afford pet ownership (RR = 0.88, 95% CI, 0.84–0.92), guardian health issues (RR = 0.91, 95% CI, 0.87–0.95), housing issues (RR = 0.94, 95% CI, 0.91–0.98), and for personal issues (RR = 0.75, 95% CI, 0.72–0.79) in comparison to surrendering for having too many animals. There was reduced risk of surrendering small animals (RR = 0.93, 95% CI, 0.89–0.97), puppies (RR = 0.86, 95% CI, 0.81–0.92), exotic animals (RR = 0.89, 95% CI, 0.81–0.97), and dogs (RR = 0.90, 95% CI, 0.86–0.93), but increased risk of surrendering kittens (RR = 1.05, 95% CI, 1.01–1.08) in comparison to cats.

Regarding health, an increase in Economic Dependency increased risk of surrendering Treatable-Rehabilitatable or Treatable-Manageable animals as opposed to Healthy animals by 1.14 (95% CI, 1.12–0.98) and 1.18 (95% CI, 1.13–1.24), respectively. This factor also predicted slightly decreased odds of surrendering an animal that is spayed/neutered upon intake (OR = 0.96, 95% CI, 0.94–0.99).

#### Residential Instability

A one-unit increase in Residential Instability increased risk of surrender for housing issues by 1.17 times (95% CI, 1.12–1.21) compared to surrendering for having too many animals. Residential Instability also increased risk of surrender for guardian health issues (RR = 1.13, 95% CI, 1.05–1.17), and being unable to afford pet ownership (RR = 1.23, 95% CI, 1.17–1.28) compared to surrendering for having too many animals. Areas higher in Residential Instability predicted increased risk of surrendering small animals and rabbits (small animals RR = 1.41, 95% CI, 1.36–1.47; rabbits RR = 1.10, 95% CI, 1.01–1.21), but decreased risk of surrendering puppies (RR = 0.60, 95% CI, 0.56–0.64), kittens (RR = 0.76, 95% CI, 0.73–0.79), and dogs (RR = 0.76, 95% CI, 0.73–0.80) compared to cats.

Residential Instability was the only CIMD factor that predicted increased risk of surrendering an animal which was Unhealthy-Untreatable compared to Healthy (RR = 1.29 95% CI, 1.21–1.37). However, it was one of two factors that predicted increased risk of surrendering animals that are spayed or neutered compared to not (OR = 1.20, 95% CI, 1.16–1.23).

### Metro Vancouver

**Figure 2:**
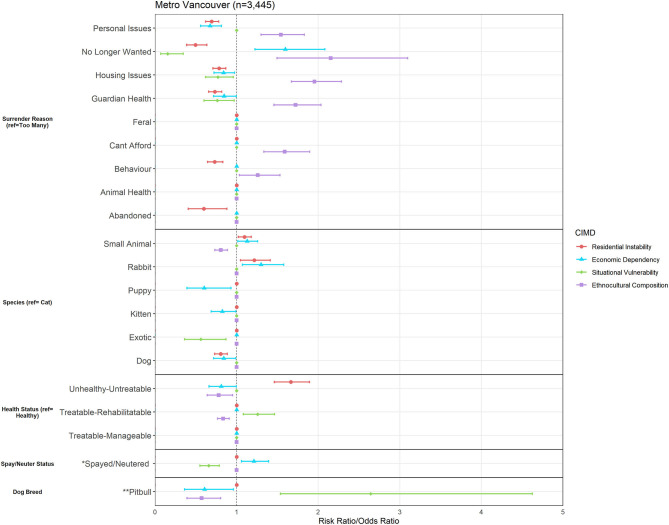
Association between Canadian Index of Multiple Deprivation (CIMD) factors and owner surrender variables across the Metro Vancouver area (*n* = 3,445). Results of logistic regression models for shelter variables by increasing CIMD factors are shown. Data are presented by risk ratios and their 95% confidence interval (bars); *p* < 0.05 when 95% confidence interval does not cross the vertical dotted line. Non-significant results are represented by a risk ratio of 1 with no CI. A risk ratio >1 suggests an increased risk of that shelter variable category as you increase in deprivation, while a risk ratio <1 suggests a reduced risk. *denotes data from dogs, cats, kittens, and puppies only (*n* = 2,128) and is represented in odds ratios (and 95% CI). **denotes data from dogs and puppies only (*n* = 609) and is represented in odds ratios (and 95% CI).

The Metro Vancouver area was subset to include any owner address that fell in between latitudes of 49.00 to 49.33 and longitudes of −123.31 to −122.46. This area includes the City of Vancouver, as well as surrounding cities and municipalities. The total number of surrenders from the Metro Vancouver area was *n* = 3,445. Results of the logistic regression models are outlined in Figure 2, which demonstrates both similarities and differences from the results of the British Columbia region. Below, we discuss relationships of interest to demonstrate descriptive examples on a geospatial scale.

**Figure 3:**
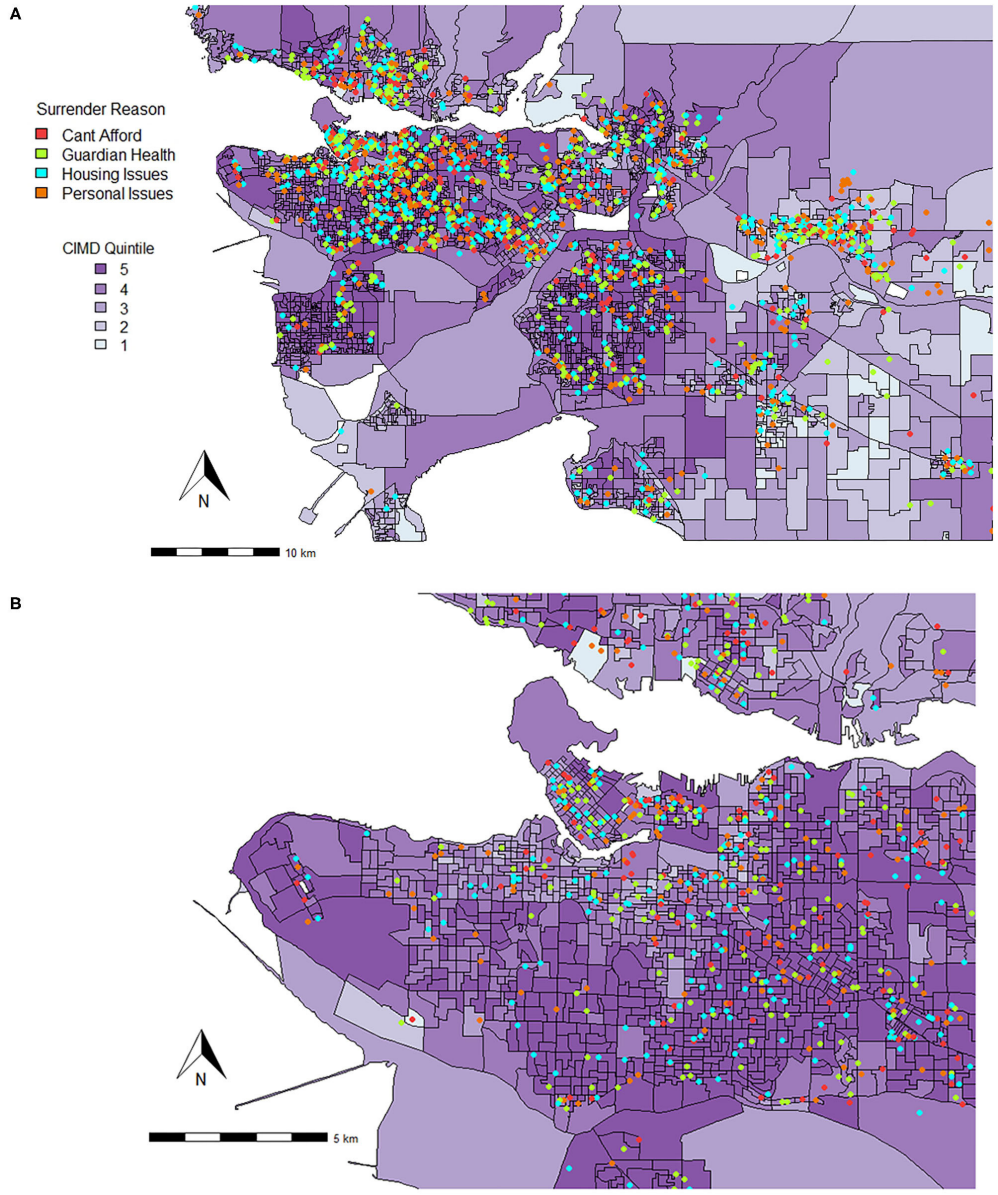
Locations where animals were surrendered for various owner-related reasons for surrender in **(A)** Metro Vancouver and **(B)** the City of Vancouver (September 1, 2016 to August 31, 2020) plotted on top of quintiles for Ethnocultural Composition (1 = least composition, 5 = most composition). The Ethnocultural Composition factor of the CIMD includes indicators such as the proportion of self-identified visible minorities; the proportion of foreign-born population; proportion of recent immigrants; and proportion of linguistically isolated population (no knowledge of either official language). While each point shows the dissemination area of origin, it does not represent the true address.

There were both similarities and differences between the relationships of CIMD and shelter variables for British Columbia and the Metro Vancouver areas. Similar to British Columbia, Ethnocultural Composition predicted increased risk of surrender for various owner related reasons such as being unable to afford the animal (RR = 1.46, 95% CI, 1.26–1.70), issues with guardian health (RR = 1.59, 95% CI, 1.39–1.83), housing issues (RR = 1.70, 95% CI, 1.50–1.93), and personal issues (RR = 1.22, 95% CI, 1.07–1.40). Owner-related reasons for surrender are plotted on a map for all of Metro Vancouver in Figure 3.

**Figure 4:**
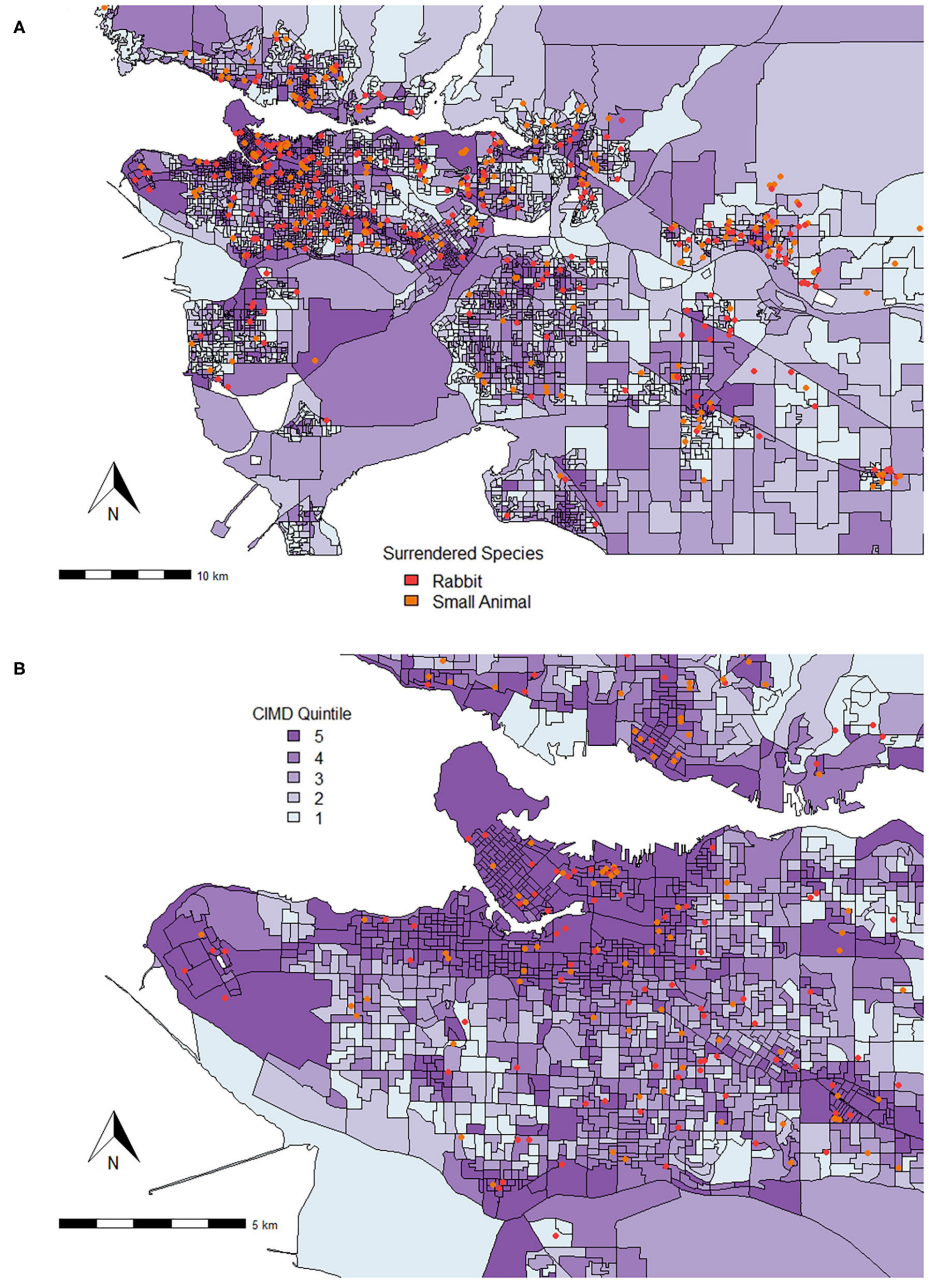
Locations where small animals and rabbits were surrendered from in **(A)** Metro Vancouver and **(B)** the City of Vancouver (September 1, 2016 to August 31, 2020) plotted on top of quintiles for Residential Instability (1 = least instable, 5 = most instablr). The Residential Instability factor of the CIMD includes indicators such as the proportion of apartment dwellings; the proportion of persons living alone; the proportion of rented dwellings; and the proportion of population that has moved in the last 5 years. While each point shows the dissemination area of origin, it does not represent the true address.

Similar to all of British Columbia, a relationship was seen between Ethnocultural Composition and decreased risk of surrender of kittens and puppies by 0.82 (95% CI, 0.69–0.99) and 0.60 (95% CI, 0.39 and 0.93) times, respectively. While increased small animal and rabbit surrender was related to Ethnocultural Composition across all of British Columbia, in Metro Vancouver, increased risk of surrender was predicted by increase in Economic Dependency for rabbits by 1.30 times (95% CI, 1.07–1.58) and small animals by 1.13 times (95% CI, 1.01–1.26) in comparison to cat surrender. Increase in small animal and rabbit surrender was also predicted by increased Residential Instability (small animal RR = 1.09, 95% CI, 1.02–1.18; rabbit RR = 1.22, 95% CI, 1.05–1.41), as seen in Figure 4.

**Figure 5:**
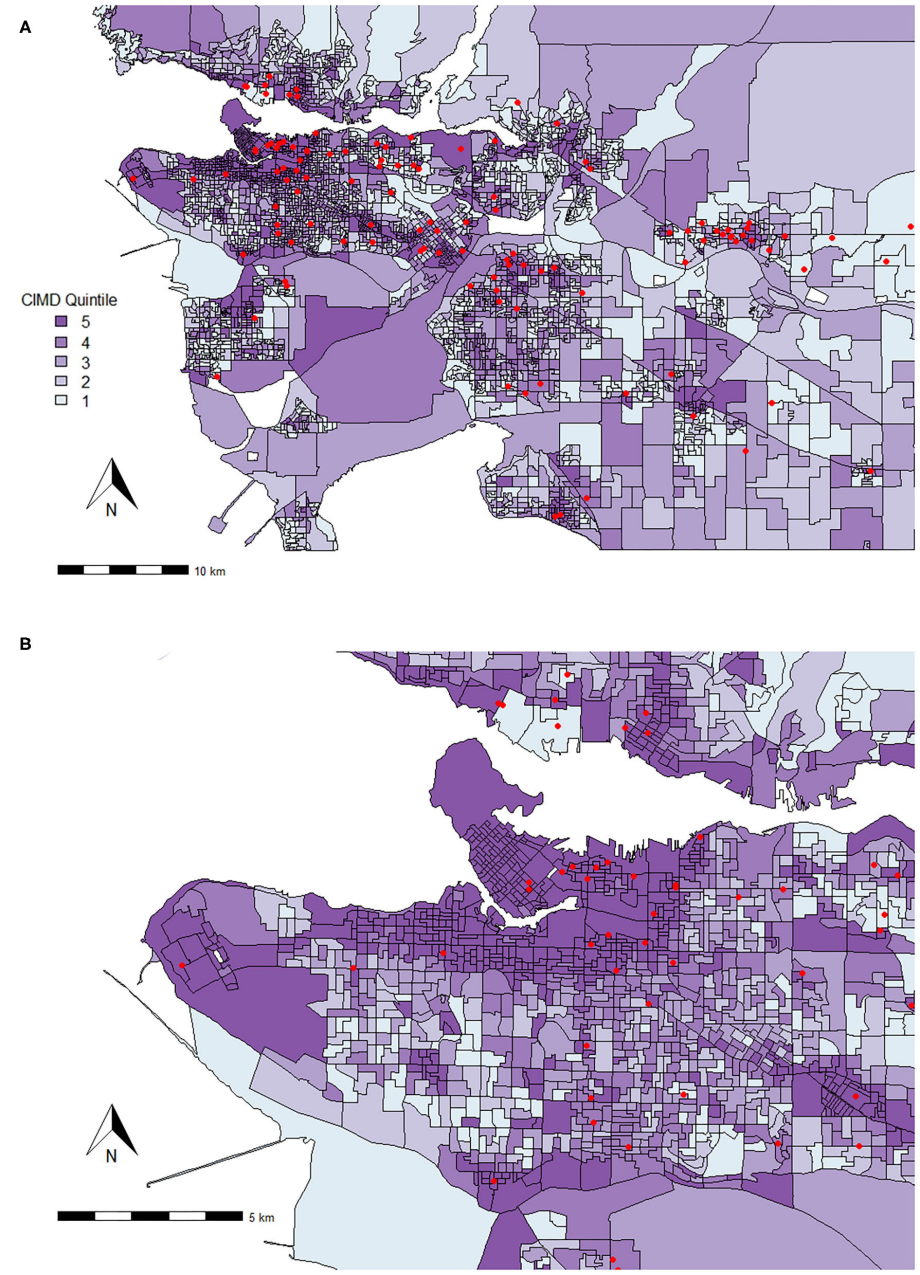
Red dots represent locations where animals scored Unhealthy-Untreatable were surrendered from in **(A)** Metro Vancouver and **(B)** the City of Vancouver (September 1, 2016 to August 31, 2020) plotted on top of quintiles for Residential Instability (1 = least instable, 5 = most instable). The Residential Instability factor of the CIMD includes indicators such as the proportion of apartment dwellings; the proportion of persons living alone; the proportion of rented dwellings; and the proportion of population that has moved in the last 5 years. While each point shows the dissemination area of origin, it does not represent the true address.

Notably, a one-unit increase in Residential Instability increased risk of surrendering an animal with an Asilomar Accords category of Unhealthy-Untreatable compared to healthy by 1.66 times (95% CI, 1.46–1.89), which is shown geospatially across Metro Vancouver in Figure 5.

Lastly, in Metro Vancouver, a one-unit increase in Situational Vulnerability increased odds of surrendering a pit bull-labeled dog by 2.6-fold (95% CI, 1.54–4.63). However, this relationship had too few points to be meaningfully visualized on a geospatial scale.

### Kamloops

**Figure 6:**
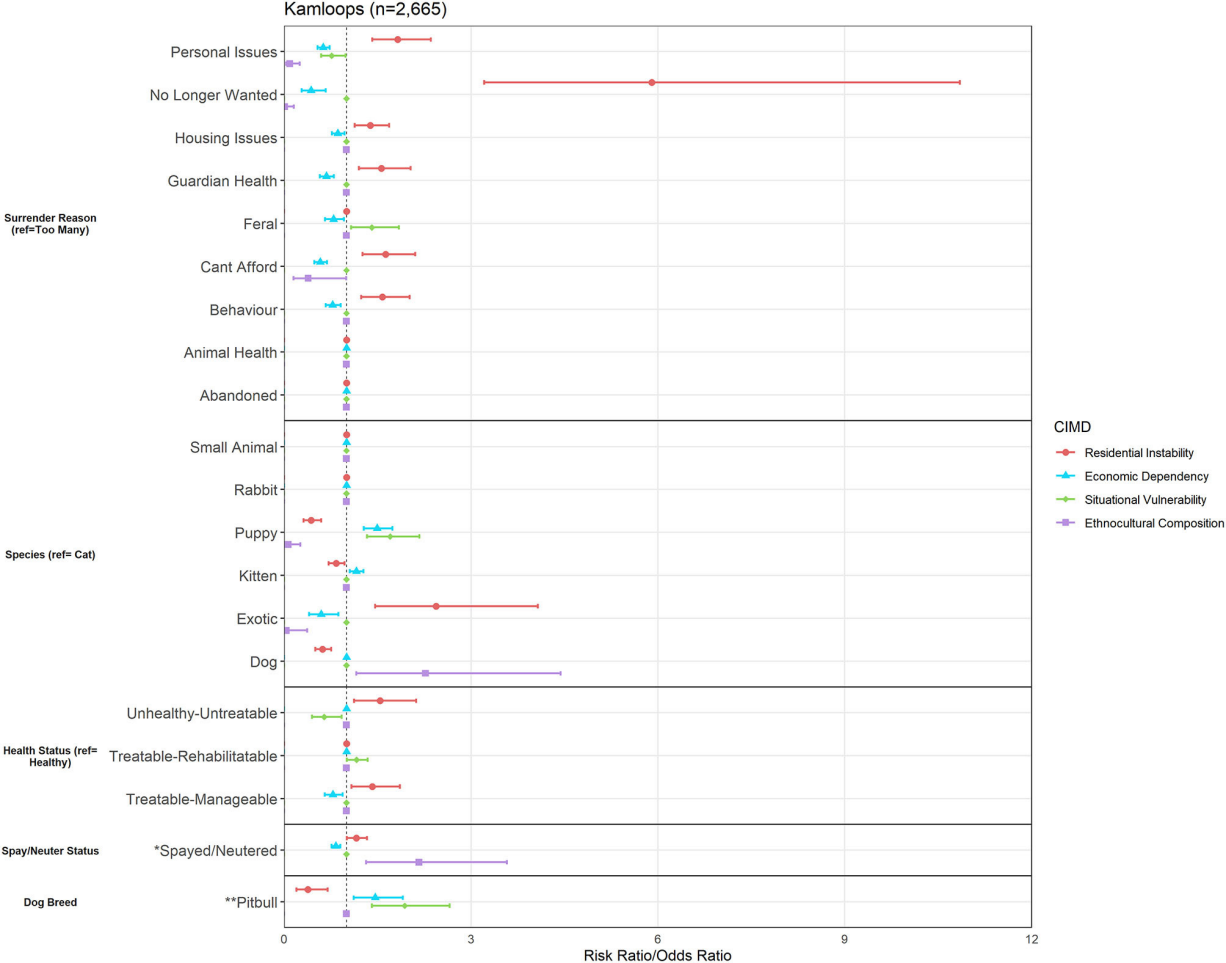
Association between Canadian Index of Multiple Deprivation (CIMD) factors and owner surrender variables across the city of Kamloops and surrounding area (*n* = 2,665). Results of logistic regression models for shelter variables by increasing CIMD factors are shown. Data are presented by risk ratios and their 95% confidence interval (bars); *p* < 0.05 when 95% confidence interval does not cross the vertical dotted line. Non-significant results are represented by a risk ratio of 1 with no CI. A risk ratio >1 suggests an increased risk of that shelter variable category as you increase in deprivation, while a risk ratio <1 suggests a reduced risk. *denotes data from dogs, cats, kittens, and puppies only (*n* = 2,548) and is represented in odds ratios (and 95% CI). **denotes data from dogs and puppies only (*n* = 657) and is represented in odds ratios (and 95% CI).

The Kamloops area was subset to include any owner address that fell in between latitudes of 50.20–51.00 and longitudes of −121.50 to −119.00. This area includes the city of Kamloops, as well as surrounding municipalities. Across all 4 years, the total number of surrenders from the Kamloops area was *n* = 2,665. Figure 6 outlines the results of the logistic regression models from the Kamloops area. Below, we discuss relationships of interest to demonstrate descriptive examples on a geospatial scale.

**Figure 7:**
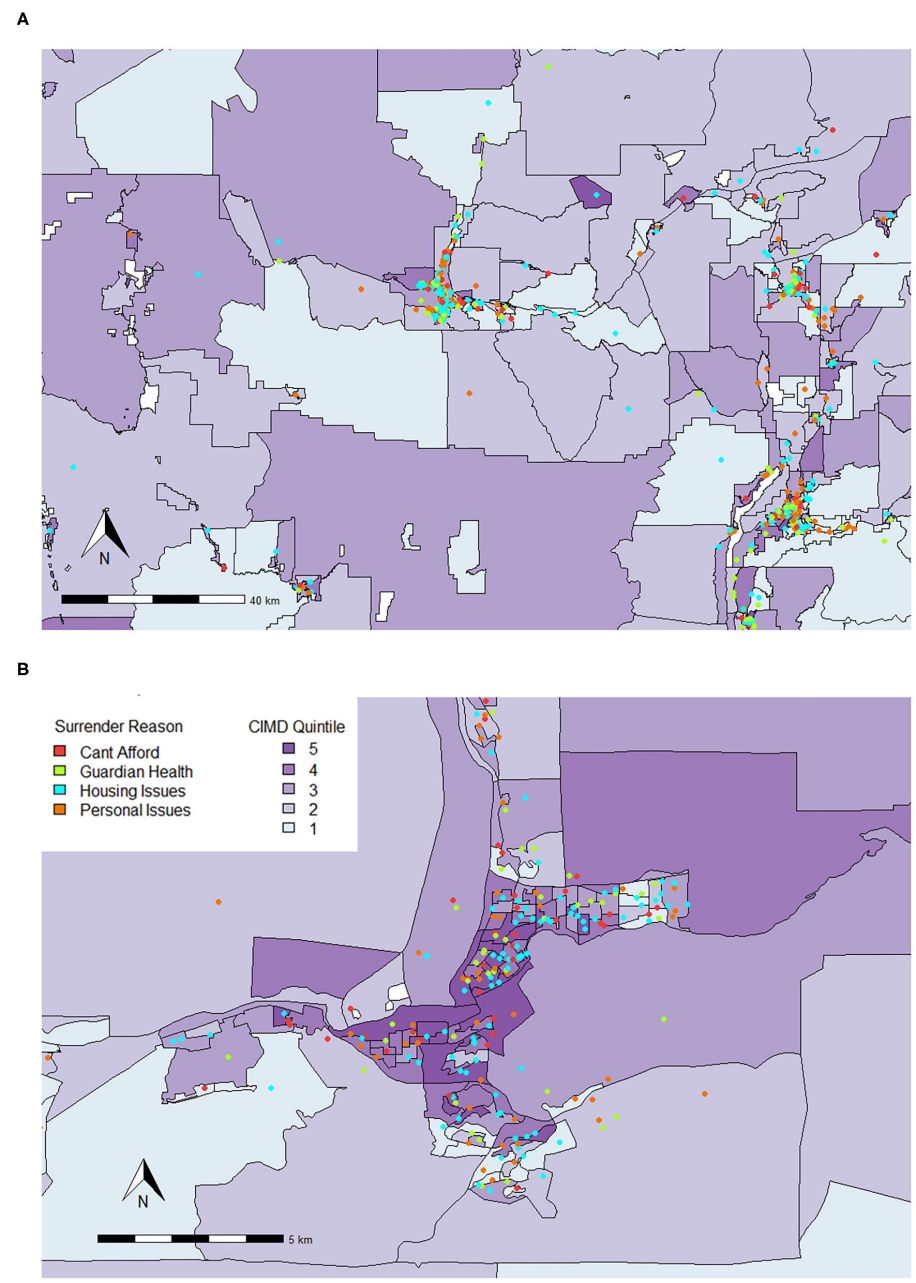
Locations where animals were surrendered for various owner-related reasons for surrender in **(A)** Kamloops and the surrounding area and **(B)** the City of Kamloops (September 1, 2016 to August 31, 2020) plotted on top of quintiles for Residential Instability (1 = least instable, 5 = most instable). The Residential Instability factor of the CIMD includes indicators such as the proportion of apartment dwellings; the proportion of persons living alone; the proportion of rented dwellings; and the proportion of population that has moved in the last 5 years. While each point shows the dissemination area of origin, it does not represent the true address.

Again, our analysis found both similarities and differences between the Kamloops area and the previously described areas of interest. In Kamloops, Residential Instability was the factor most related to various owner-related reasons for surrender, as it predicted increased risk of surrendering animals for personal issues by 1.82 times (95% CI, 1.41–2.35), housing-related issues by 1.38 times (95% CI, 1.13–1.69), being unable to afford the animal by 1.63 times (95% CI, 1.26–2.10), and guardian health issues by 1.56 times (95% CI, 1.20–2.03), in comparison to the reason of having too many animals. These owner-related reasons are plotted geospatially across the Kamloops area in Figure 7.

Unlike British Columbia and Metro Vancouver, there was no relationship between any of the CIMD factors and risk of surrendering small animals and rabbits. Both Situational Vulnerability and Economic Dependency predicted increased risk of surrendering puppies by 1.70 (95% CI, 1.33–2.17) times and 1.49 (95% CI, 1.28–1.74) times, respectively (in comparison to cats). Residential Instability predicted decreased surrender of puppies (RR = 0.43, 95% CI, 0.31–0.60) and kittens (RR = 0.83, 95% CI, 0.71–0.97), but increased risk of surrendering exotic animals by almost 2.5 times (RR = 2.43, 95% CI, 1.46–4.07).

**Figure 8:**
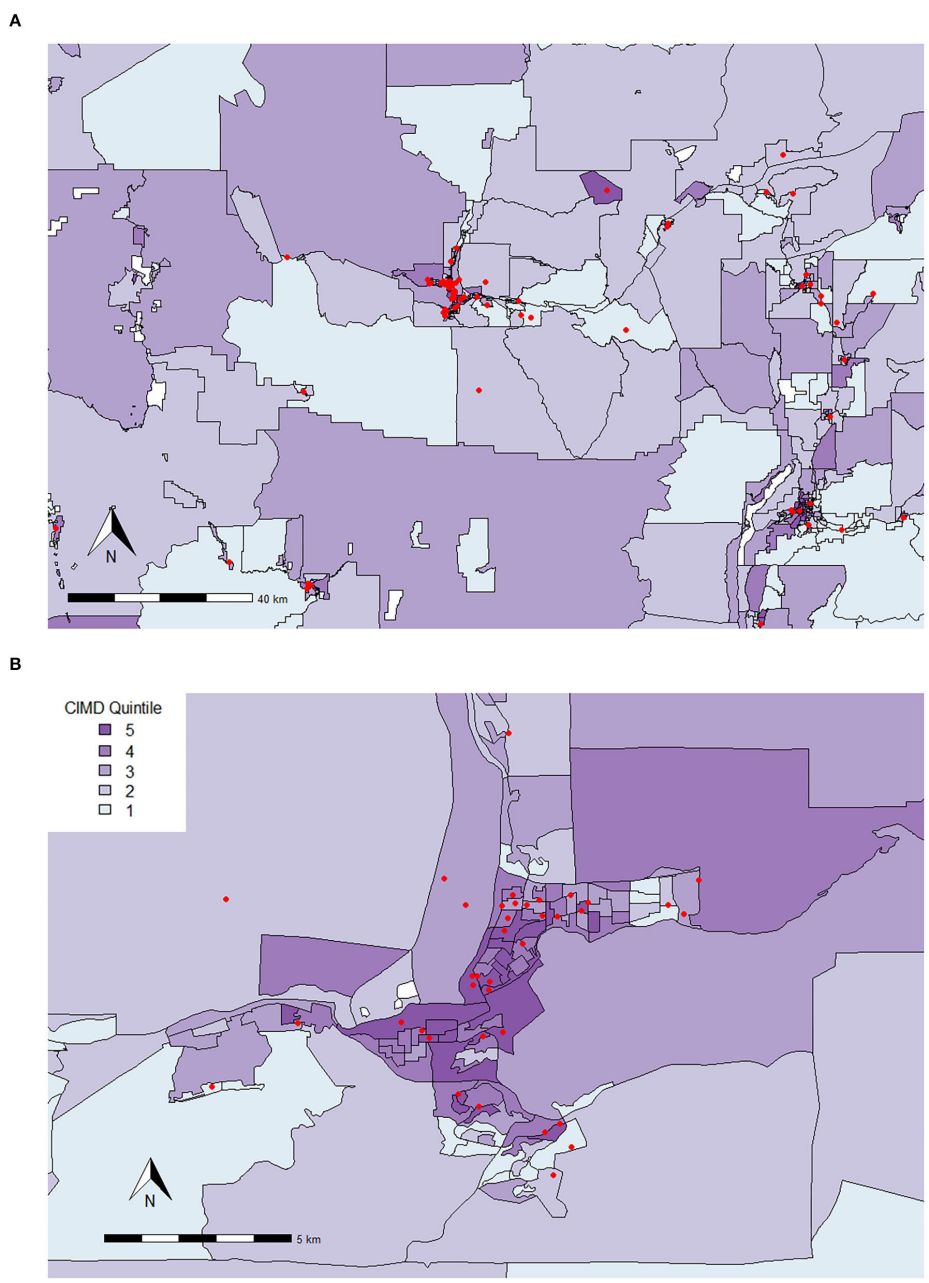
Red dots represent locations where animals scored Unhealthy-Untreatable were surrendered from in **(A)** Kamloops and surrounding areas and **(B)** the City of Kamloops (September 1, 2016 to August 31, 2020) plotted on top of quintiles for Residential Instability (1 = least instable, 5 = most instable). The Residential Instability factor of the CIMD includes indicators such as the proportion of apartment dwellings; the proportion of persons living alone; the proportion of rented dwellings; and the proportion of population that has moved in the last 5 years. While each point shows the dissemination area of origin, it does not represent the true address.

Similar to Metro Vancouver, a one-unit increase in Residential Instability predicted increased surrender of Unhealthy-Untreatable animals by 1.54 times (95% CI, 1.12–2.11) compared to surrender of Healthy animals. For comparison with the Metro Vancouver area, this relationship is plotted across the Kamloops area in Figure 8.

Another similarity between Kamloops and Metro Vancouver was related to pit bull-labeled dogs. A one-unit increase in Situational Vulnerability increased odds of surrendering a pit bull-labeled dog by almost 2-fold (OR = 1.94, 95% CI, 1.41–2.66). As with Vancouver, there were too few points to represent this meaningfully on a geospatial scale.

## Discussion

Our study found multiple relationships between human deprivation and animal relinquishment across British Columbia. Exploratory analysis of multiple human and animal related factors allowed us to assess social determinants impacting animal relinquishments on a broad scale, which can be used to inform further research in areas of human and animal welfare. The present study is among the first to explore owner surrender data based on human vulnerability, and part of the growing literature using geospatial analysis to visualize these relationships on a community scale.

Our study found that higher Ethnocultural Composition scores predicted increased surrender for multiple owner related reasons, including guardian health issues, housing issues, personal issues, and being unable to afford the pet. Racialized and immigrant populations have been previously linked to these reasons for surrender. The term racialized is used in Canada to replace terms such as “visible minority,” in recognition that race is a social (not biological) construct and that in some parts of the country, former “minority” populations now comprise a majority (27). For example, Canadians that identify as an ethnic minority are shown to be at higher risk of various poor physical and mental health variables (28, 29). Immigrants to British Columbia report difficulties in finding housing due to unfamiliarity with the Canadian housing system and unexpectedly high costs (30). Vancouver neighborhoods with more homogenous minority populations were found to have higher prices (31), which possibly puts racialized populations at higher risk of housing-related surrender issues. Indeed, racialized and immigrant populations may face adversities that increase risk of companion animal surrender for owner-related issues.

Furthermore, while previous studies often investigated surrender of dogs or cats, our results indicate that further investigation is needed among all species and age groups. Greater Ethnocultural Composition increased risk of surrendering kittens, puppies, rabbits, and small animals compared to adult cats. Studies surrounding differences in race or ethnicity and animal ownership are lacking, particularly with different species of companion animals. Applebaum et al. (32) reported 70.4% of White Americans owned pets compared to 29.0% of African Americans, 60.0% of Latin Americans, and 33.0% of all other races/ethnicities. Previous studies have found variation in attitudes toward animals and animal ownership based on cultural or ethnic group membership (33, 34). A Dutch study found that self-identified ethnic minorities kept pets less frequently, and owned atypical companion animals such as fish and birds more frequently (35). However, it would be relevant to further investigate cultural differences of pet ownership in Canada, since differences in deprivation are expected based on different composition of the ethnic populations compared to the United States [such as lower population of Latin Americans in Canada; (36)]. While pet ownership occurs in virtually all cultures globally, it is relevant to explore the differences in experienced barriers between cultures in order to better understand relinquishment of animals from racialized households and provide useful support.

The Situational Vulnerability dimension of the CIMD represents variation in socio-demographic conditions across education, income, and housing. In British Columbia, indicators that contribute to Situational Vulnerability included low educational attainment; low income; single parent families; housing needing major repair; and proportion of Indigenous population. Previous studies have assessed the relationship between income and pet ownership. For example, a Brazilian survey found that higher income households were more likely to own dogs compared to lower income households, but there was no difference in likelihood of cat ownership or number of animals owned (37). A study in the Netherlands found that animal owners tended to have higher incomes than non-owners (38). Our study found that Situational Vulnerability in British Columbia was related to various risks, such as surrendering litters (kittens or puppies). The relationship between income and unwanted litters has previously been investigated, and has led to the creation of low-cost spay/neuter clinics (39).

The Situational Vulnerability factor also includes the proportion of Indigenous peoples in a dissemination area. Indigenous peoples face higher rates of social and health deprivation including increased poor health variables, poverty, inadequate housing, and discrimination (40). Research shows that companion animals are culturally important for Indigenous communities, and pet ownership plays an important role in the social facilitation of Indigenous youth in Canada (41). However, underserved Indigenous communities are also at higher risk of zoonotic diseases from domestic dogs, and many struggle with dog population management due to rural locations and lack of access to veterinary care (42). There are some Indigenous groups for which veterinary medical procedures (e.g., surgically altering dogs, vaccination, and deworming) are not culturally acceptable (42). As well, Indigenous communities are not homogenous in nature with relation to animal ownership (43). An estimated 44% of Canada's Indigenous population resides in urban areas (44). Therefore, it is possible that issues with access to veterinary services are not always geospatial, but perhaps temporal or financial, as examples. Issues with Indigenous access to care may have been reflected in our study, as Situational Vulnerability was associated with population issues such as surrendering for having too many animals, surrendering litters, and surrendering sexually intact animals.

The Economic Dependency dimension is related to dependence on sources of non-employment income. This includes measures such as the proportion of population aged 65 and older, unable to work (<15 years old), and the proportion of the population that is unemployed. Economic dependency also indicates presence of two possible age groups—adolescents and seniors. From a health perspective, pet ownership in adolescence has been shown to improve psychosocial development [for a review, see (10)]. In seniors, pet ownership can improve loneliness, social engagement and physical activity, among other benefits [for a review, see (45)]. The aging population also faces unique difficulties with pet ownership, such as being unable to bring their pet to care homes or having to find care for the pet while dealing with health issues (46). However, our study was not congruent with previous findings, as Economic Dependency reduced risk of surrendering for owner-related reasons compared to having Too Many animals.

Although the Economic Dependency dimension deals with mainly employment-related measures, increase in Economic Dependency predicted *decreased* risk of surrendering for reasons of being unable to afford the pet. However, across British Columbia, an increase in Economic Dependency predicted increased risk of animals that were not considered healthy under the Asilomar Accords (Treatable- Rehabilitatable and Treatable-Manageable) and also predicted increased risk of surrendering intact animals. Risk of poor health status and sexually intact animals may also be related to cost, as price/cost has been cited as a reason animal owners do not seek more frequent veterinary care (47).

The Residential Instability dimension encompasses neighborhood fluctuations in terms of both housing and familial factors. Residential Instability predicted increased risk of surrender due to housing issues, which has been cited as a common issue motivating animal relinquishment (11), including with animals other than dogs and cats [e.g., rabbits; (48)]. Factors of Residential Instability have previously been identified as issues in pet ownership [such as being a renter; (49)]. The rising cost of home ownership has led to increased numbers of renters, and in many areas, landlords have significant control of the type, number or size of animals in rental units (50). Accommodating pets when renting means that some owners choose lower quality or higher priced rentals, or even keep pets without approval (51). Housing-related worries, such as living in unsuitable accommodations for a pet, living in an unsafe neighborhood for a pet, or living in a no-pets building, are reported as some of the most significant reasons of non-ownership of pets (8, 52). Indeed, because many pet owners see their animals as “family” (53), the issues of housing and pet ownership are inextricably linked to each other.

Analysis of the Vancouver and Kamloops regions shows that there are geographic differences in the predictive nature of human deprivation on owner surrender factors. These geographic differences are reflected in different CIMD scores between locations. For example, Ethnocultural Composition may differ significantly based on the area. While immigrants prefer to settle in major metropolitan cities (such as Vancouver), this has led immigration policies to encourage immigrants to settle in smaller cities [such as Kamloops; (30)]. This was reflected in our study as Vancouver showed high Ethnocultural Composition scores across most dissemination areas. Metro Vancouver is more urban compared to the area of Kamloops, and pet ownership is greater in rural areas compared to urban (8).

There are also differences between Vancouver and Kamloops that are not directly captured by the CIMD, but may impact pet ownership and surrender. One example of this is the built environment of the cities. In Canada, municipal governments are generally responsible for jurisdiction of park development, including dog parks (54). Previous studies in Canada have found that proximity of off-leash dog parks in a neighborhood impacts dog-walking behavior, which can impact health status of both the owner and the dog (55, 56). However, research shows that built environments of neighborhoods are linked to deprivation factors such as socioeconomic status and racial or ethnic composition (57, 58), so this is likely captured by a similar relationship to CIMD factors.

Our results also showed factors which similarly predicted shelter surrender variables between all three areas of interest. For example, Situational Vulnerability predicted increased odds of surrendering pit bull-labeled dogs compared to all other dog breeds. The term “pit bull” does not correlate to a recognized breed, but instead is a term applied to a heterogeneous group of purebred and mixed breed dogs, although the use of pit bull as a descriptor varies according to observers (59), and dogs are often mislabeled in shelters (20). Despite common visual misidentification, many provinces and states impose Breed Specific Legislation (BSL) that restricts the ownership of pit bull-labeled dogs based on fear of injury to humans (60). Pit bull-labeled dogs have long been associated with racialized populations and poverty (19). Media often shows pit bull-type dogs in relation to aggression and dog fighting (61). While dog fighting originated in Britain, the image of dog fighting in the United States is associated predominantly with low-income Black men (62). The animal welfare field has long had suspicions that BSL is more linked to racism rather than public safety through the transfer of racial stereotypes and stigma [c.f. (63, 64)]. Our findings provide support for the relationship between the relinquishment of pit bull-labeled dogs and socioeconomic status, as Situational Vulnerability was associated with increased risk of surrender for dogs labeled this way by shelter staff.

Companion animal ownership is common, and research has explored the mental and physical health benefits in humans who own companion animals. Several studies compare health variables for companion animal owners and non-owners and find that those with companion animals have improved health variables such as decreased mental health issues (65), lower risk of heart disease (66) and increase in positive health behaviors (67). Research has found particular benefits of pet ownership in vulnerable populations. For example, youth experiencing homelessness reported fewer symptoms of depression if they owned a companion animal (68). Furthermore, animal relinquishment is a difficult and emotional decision for animal owners (69). Because vulnerable populations may be more at risk of surrendering animals, addressing deprivation could reduce surrender of animals from populations whose health may benefit greatly from pet ownership, as well as reduce the stress associated with the decision to relinquish animals among this population.

The results in our study also indicate that deprivation may be related to lack of access or other barriers in seeking services for companion animals. All four factors were related to risk of either not spaying/neutering the animal or the animal having a health status other than Healthy upon intake. This is in line with previous research in Great Britain, which found that dogs and cats belonging to owners in more deprived areas were less likely to have preventative health care interventions such as being spayed/neutered and being microchipped (70). However, surrendering the animal for reasons of the animals' health was not related to any of the factors of CIMD, demonstrating that these barriers may be experienced but may not be the primary cause of surrender. Reasons such as cost or lack of a regular veterinarian may be barriers to seeking veterinary care (39). Indeed, improving access to veterinary care for low-income and other vulnerable pet owners has been identified as one of the main challenges of veterinarians in Canada (71). There is also an argument to improve access to veterinary care from a public health perspective, which is commonly argued from a One Welfare perspective. For example, zoonotic disease transmission via pets can cause health and economic costs for both humans and animals (72). Underserved communities are less likely to have regular visits with a veterinarian, and thus are less informed about zoonotic disease risks (73). The literature regarding access to veterinary care parallels the research in human healthcare, which perhaps indicates a One Welfare connection for families with companion animals. For example, increased risk of oral disease in children is associated with race (e.g., Black and Mexican Americans) and income (living below the poverty level) in the United States (74). The similarities between lack of access to veterinary care and health care demonstrate the impact of deprivation on human and non-human animal family members. Further research could investigate access to both human and animal services in communities to determine the impact of multiple deprivations on families.

Due to the relationships between human deprivation and lack of available animal-related services, the results of our study support improving provision of services to both humans and companion animals to decrease risk of surrendering for deprivation-related reasons. Examples of services which address both human and animal welfare have shown success. For example, households facing food insecurity reported highly valuing having pet food available in their food banks, and were reluctant to surrender animals if this food became unavailable, instead agreeing that they would share human food with their companion animals (75). As previously mentioned, the implementation of free or subsidized spay/neuter clinics both alleviates cost-related concerns of veterinary care (76), but can also improve welfare of the animal, as previous studies have found that spay/neuter clinics can reduce rates of intake in shelters (77). As well, the evidence of pet ownership and health benefits in aging adults has led to the development of programs like the TigerPlace Pet Initiative (TiPPI), a senior living facility that is pet-inclusive and provides veterinary care, foster care, and adoption services for the residents (78). Weiss et al. (79) found that dog owners reported that interventions such as low-cost dog support (e.g., training or veterinary care), temporary boarding, and pet-friendly housing may have helped them retain their pets. These initiatives follow principles of the One Welfare framework, as providing services for the animals ultimately improve human well-being through retention of a companion animal, reduction of stress related to costs of owning an animal, or reducing risk of zoonotic disease transmission (6). Our study also shows that owner-related deprivations increase risk of surrendering animals to shelters, indicating a need for owner-related interventions to keep pets in their homes. However, our study identified other human-related social issues in risk of relinquishment to shelters, such as ethnicity, housing insecurity, and Indigenous status which would benefit greatly from further research to identify appropriate support interventions.

There are some notable limitations to our study. The shelter data were limited to a single reason for surrender, although relinquishment of pets has been identified as a complex and multifaceted decision (80). Previous studies found that owners may misreport reasons for surrender when undergoing the difficult decision to relinquish their animal due to social desirability bias (81). For example, an owner may report a behavioral issue in the animal to avoid admitting being unable to take care of the animal. Similarly, shelter staff may report reasons for relinquishment differently based on location, based on internal bias of staff members, or based on differing definitions between the animal owner and staff. This limitation has been identified in previous literature (11, 81), although it may be more relevant as our data was taken across multiple shelter locations with varying staff.

One possible limitation of using the CIMD is that it reflects deprivation relative to the pet owners' location rather than the current status of the individual owner. However, some of the factors used to calculate individual dimensions, such as proportion of apartment dwellings, are characteristics of the dissemination areas that are calculated independent of individual socioeconomic status. Furthermore, the CIMD is calculated in the smallest unit of area for which all census data is collected, meaning this data is the most specific form of measurement of social vulnerability for geospatial comparison across the whole province. Thus, we believe that the CIMD is a valuable proxy to represent general socioeconomic status for the individuals, as it is likely that individuals living in the same area share similar factors of deprivation (70).

Finally, although the CIMD represents social facets that are likely to relate to vulnerability of communities, some factors leave more room for interpretation than others. For example, the Economic Dependency factor uses indicators that include age demographic of an area, including those who are over 65. Economic Dependency could be high in retirement communities with a high population of over 65 residents (who may be affluent), or high in other communities with families that have children that are <15 years old. Our study found that, across British Columbia, Economic Dependency predicted decreased risk of surrender for multiple owner-related reasons for surrenders, which could be due to the indicators of this CIMD factor. For further research, it may be helpful to distill the more straightforward elements of the CIMD, or more specific census data of interest (e.g., income, rental housing) that have *a priori* predictions related to animal surrender.

## Conclusion

Our analysis showed that, from 2016 to 2020, various dimensions of the Canadian Index of Multiple Deprivation (CIMD) were related to risk of animal relinquishment based on animal shelter variables such as owner-related reasons for surrender, species, health status, and breed, to the British Columbia Society for the Prevention of Cruelty to Animals. The use of geospatial analysis identified differences in deprivation and animal relinquishment risks for different geographic areas within the province of British Columbia. Identifying CIMD dimensions which are associated with shelter intake data does not necessarily imply a causal association. However, the results of this study can be used to inform shelter or community-based interventions. Viewed through a One Welfare lens, people experiencing vulnerability benefit from continued pet ownership—supporting retention of animals and understanding factors that lead to surrender is valuable at both the shelter and community level. To reduce relinquishment of animals to shelters and to keep animals with their owners, a focus on identifying and providing support for vulnerable populations is relevant.

## Data Availability Statement

The original contributions presented in the study are included in the article/Supplementary Material, further inquiries can be directed to the corresponding author/s.

## Ethics Statement

The studies involving human participants were reviewed and approved by University of British Columbia Behavioral Research Ethics Board. Written informed consent for participation was not required for this study in accordance with the national legislation and the institutional requirements.

## Author Contributions

LL, EG, and AP contributed to the conception of the study and subsequent study design. LL acquired and organized the database and wrote the first draft of the manuscript. LL and AP performed statistical analysis. All authors contributed to the manuscript, further revisions, and approved the final version of the manuscript for submission.

## Conflict of Interest

Data was obtained from the BC SPCA, who is the employer of one of the authors EG. The remaining authors declare that the research was conducted in the absence of any commercial or financial relationships that could be construed as a potential conflict of interest.

## Supplementary Material

Supplementary Data Sheet 1 Ly et al. Code. Github link for access to R code used to analyze the relationship between human deprivation and animal surrender in British Columbia.

Supplementary Data Sheet 2 Ly et al. Data File. Owner surrender to BC SPCA shelters from September 1, 2016 to August 31, 2020.
