# Supplementary material for: Exploring the Relationship Between Human Social Deprivation and Animal Surrender to Shelters in British Columbia, Canada
Source: Front Vet Sci. 2021 Mar 10;8:656597. doi: 10.3389/fvets.2021.656597 (PMC8006318; doi:10.3389/fvets.2021.656597)
Supplement: Supplementary Data Sheet 1 — Ly et al. Code. Github link for access to R code used to analyze the relationship between human deprivation and animal surrender in British Columbia. [file Data_Sheet_1.DOCX]

Access to R Code for “Exploring the relationship between human social deprivation and animal surrender to shelters in British Columbia, Canada”

https://github.com/lexisly/deprivation-surrender
